# Design of a dietary intervention to assess the impact of a gluten-free diet in a population with type 1 Diabetes and Celiac Disease

**DOI:** 10.1186/s12876-015-0413-0

**Published:** 2015-12-21

**Authors:** Esther Assor, Margaret A. Marcon, Natasha Hamilton, Marilyn Fry, Tammy Cooper, Farid H. Mahmud

**Affiliations:** Department of Pediatrics, Division of Endocrinology, Hospital for Sick Children, University of Toronto, Toronto, ON Canada; Division of Gastroenterology, Hepatology and Nutrition, Hospital for Sick Children, University of Toronto, Toronto, ON Canada; Division of Endocrinology Paediatrics, London Health Sciences Centre, London, ON Canada; Division of Endocrinology, Markham Stouffville Hospital, Toronto, ON Canada; Division of Endocrinology and Metabolism, Children’s Hospital of Eastern Ontario, Ottawa, ON Canada

## Abstract

**Background:**

Celiac Disease occurs at a 5–10 fold greater prevalence in patients with type-1 diabetes (T1D), despite this increased risk, there is limited objective evidence regarding the impact of a Gluten-Free Diet (GFD) in the large proportion of asymptomatic (30–70 %) patients with both autoimmune diseases. Given the requirements and intricacies inherent to each condition, we describe the rationale and design a dietary curriculum specifically addressing the educational requirements for children and adults with CD and diabetes as part of the CD-DIET Study.

**Methods and design:**

The CD-DIET Study (Celiac Disease and Diabetes - Dietary Intervention and Evaluation Trial) is a multicenter randomized controlled trial aimed at evaluating the safety and efficacy of a GFD in patients with asymptomatic celiac disease and T1D on key diabetes and patient-centered outcomes.

**Discussion:**

Key dietary components of the trial include a description and evaluation of food consumption patterns including glycemic index and glycemic load, novel assessments of gluten quantification, and objective and subjective measures of GFD adherence. This dietary curriculum will establish rigorous guidelines to assess adherence and facilitate evaluation of a GFD on metabolic control, bone health and patient quality of life in patients with CD and diabetes.

**Trial registration Number:**

NCT01566110. Date of Registration: March, 2012.

## Background

Celiac Disease (CD) is an autoimmune disease characterized by damage to the small intestine, triggered by gluten ingestion from wheat, rye and barley and their derivatives in genetically susceptible individuals [1, 2]. Currently the only treatment for CD is a lifelong adherence to the gluten-free diet. CD prevalence in T1D patients approximates 5-7 %. Despite this increased risk, many health care professionals struggle as to the optimal approach to managing CD in Type 1 Diabetes (T1D). The diagnosis of patients who present with symptomatic CD, including malabsorption and obvious pathology upon biopsy, remains straightforward, with improvements noted on a gluten-free diet. Many patients identified by screening, however, tend to be asymptomatic [3, 4]. Evidence is inconclusive as to whether the benefits of screening and potentially treating asymptomatic individuals outweigh the harms of managing a population already burdened with an established chronic illness.

**Fig. 1:**
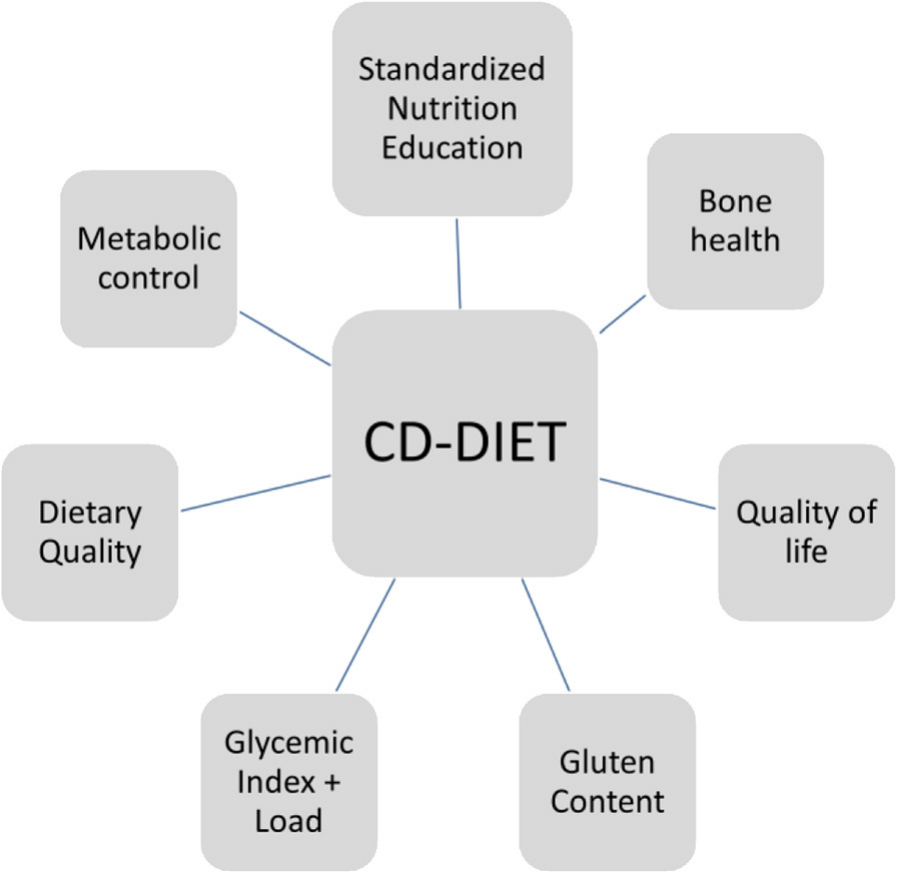
Primary and Secondary Outcomes for CD-DIET (Celiac and Diabetes-Dietary Intervention and Evaluation Trial)

The CD-DIET Study (Celiac Disease and Diabetes - Dietary Intervention and Evaluation Trial) is a multicenter randomized controlled trial aimed at evaluating the safety and efficacy of a GFD in patients with asymptomatic celiac disease and T1D over 1 year. Key outcomes include the impact of GFD on diabetes control (HbA1c), bone health, blood glucose variability, and patient centered measures including quality of life and self-perceived health. Dietary outcomes will include description and evaluation of food consumption patterns including glycemic index and load, dietary characteristics including gluten quantification, and highlight objective and subjective measures of GFD adherence. As a consequence, a de-novo dietary curriculum specifically addressing the educational requirements for GFD and T1D was developed to study dietary adherence and facilitate evaluation of key dietary components and their impact on study outcomes (Fig. 1).

An additional challenge of this trial relates to potential difficulties with individual adherence to their assigned dietary group. As such, significant effort was invested in a patient and family centered dietary experience supported by dietitians with extensive knowledge of diabetes, and a curriculum with subjective and objective assessment for dietary quality and gluten content. A de novo study dietary handbook was also created for the study, with sections focusing on adaption to the GFD highlighting situational approaches and providing gluten-free diabetes-specific nutritional tables for carbohydrates and glycemic index. This paper describes approach and details of the dietary curriculum implemented as part of the CD-DIET study to address the needs and challenges of living with asymptomatic CD and T1D.

### The challenge of T1D & CD: dietary quality and education

CD is a unique condition since dietary modification is the only currently recommended and effective treatment. Type 1 diabetes is a challenging condition requiring daily efforts to balance meals, activity, and insulin to maintain adequate metabolic control. It is important to assess the impact of CD on dietary quality and composition as transition to a GFD may pose a risk of nutritional imbalances and/or deficiencies. Dietary inadequacies have been reported to be common with CD. This may be inherent to the diet but also as a result of habitual poorer choices [5–7], although it has been shown that imbalances from the GFD are no different than the imbalances noted in the healthy population [8]. Gluten-free foods available in Canada, analyzed for macro/micronutrient composition were found to be lower in fiber, iron, calcium, protein, folate and vitamin D and higher in total carbs, fat and sugar [5, 6, 9, 10] than products containing gluten. Similar imbalances including higher fat, lower fiber and nutrient intakes have been noted in children and adults with T1D [11, 12]. Compared to their gluten containing equivalents, processed GF foods including breads, cereal bars and pasta have been reported to be higher in total carbohydrates, lower in protein and higher in sodium, saturated fat and cholesterol [13]. Additionally, many starchy GF foods were noted to have variable mineral content and high glycemic index [14]. From a Canadian context, both the Canadian Celiac Association (CCA) and the Canadian Diabetes Association (CDA) offer access to pertinent information including nutritional and financial support strategies for each medical condition. However there are limited all-encompassing dietary advice/resources catered to needs of individuals living with the double diagnosis of CD and T1D. A comprehensive literature review including PubMed, Medline, University of Toronto Libraries and various Internet websites investigated what resources are available on the subject. Our research found that the current available literature is limited in providing effective meaningful and tangible resources, tailored to address the short and long term needs and challenges of both conditions, particularly of asymptomatic patients with CD and T1D. At our center we have a unique experience of having the opportunity to follow this population affected by a double diagnosis of Type 1 diabetes and Celiac Disease, combined with an expertise and specialization in this field. A comprehensive curriculum has been developed to address the perceived and noted challenges as practitioners, patients and the literature.

### Impact of CD in T1D: Quality of life (QOL)

In studies of existing chronic conditions such as diabetes, which involve mandatory daily routines to remain safe, and daily challenges which require problem solving, it is important to evaluate the impact of the double diagnosis (CD and T1D) that may further negatively impact quality of life (QOL). Areas of negative impact in maintaining a GFD have been described as follows: cost maintaining a GFD, and extend to socializing GF including dining out, impact on family, career, travel and religious practices [15–18] and the corresponding negative psychological symptoms and negative emotions [19, 20]. The benefit of the gluten-free diet (GFD) on QOL in asymptomatic screened-detected CD patients remains controversial [20–23]. Asymptomatic patients with CD report better self-perceived health and less concern with their disease prior to dietary modification [24, 25], and over half of patients following a GFD for more than 5 years have reported QOL as excellent or very good [26].

Given these challenges and our clinical experience with our population, a key element of the dietary curriculum is to address all the challenges through education. Information is provided in a supportive, encouraging and individualized manner with regular visits where patients are asked to bring in labels and practice problem solving. The Book’ Your Guide to Well Being: Managing Celiac Disease and Diabetes’ is written in a modular form so that patients and their families could refer to various sections that they find pertinent. The book addresses and provides tangible and concrete guidance on living with Type 1 and CD. It addresses in detail all aspects of social life and provides practical advice for incorporating a GFD while eating out, traveling, celebrations, management at school, university, and the workforce. A resource list including support groups, helpful websites, associations and organizations, a detailed list of GF foods with their macro and micronutrient contents including GI is available to support the practical day to day life of CD and T1D. To limit financial barriers, all patients on a GFD are provided with a monthly stipend for their groceries.

### Dietary assignment in a clinical trial: opportunities and challenges

Symptomatic patients who follow a GFD experience an improvement in symptoms and therefore following a GFD is meaningful. However it is less clear if there are any related benefits to asymptomatic CD patients with T1D who follow a GFD. The CD-DIET Study represents an opportunity to evaluate the impact of a GFD on asymptomatic patients with CD. This is a randomized, controlled, multicenter, dietary intervention study in pediatric and adult diabetes centers across Ontario, Canada, that is based on an intention to treat. The study consists of a comprehensive dietary interview with defined subjective and objective parameters. It is designed to address the educational and psychosocial needs and challenges of living and thriving with both conditions: CD and T1D.

**Fig. 2:**
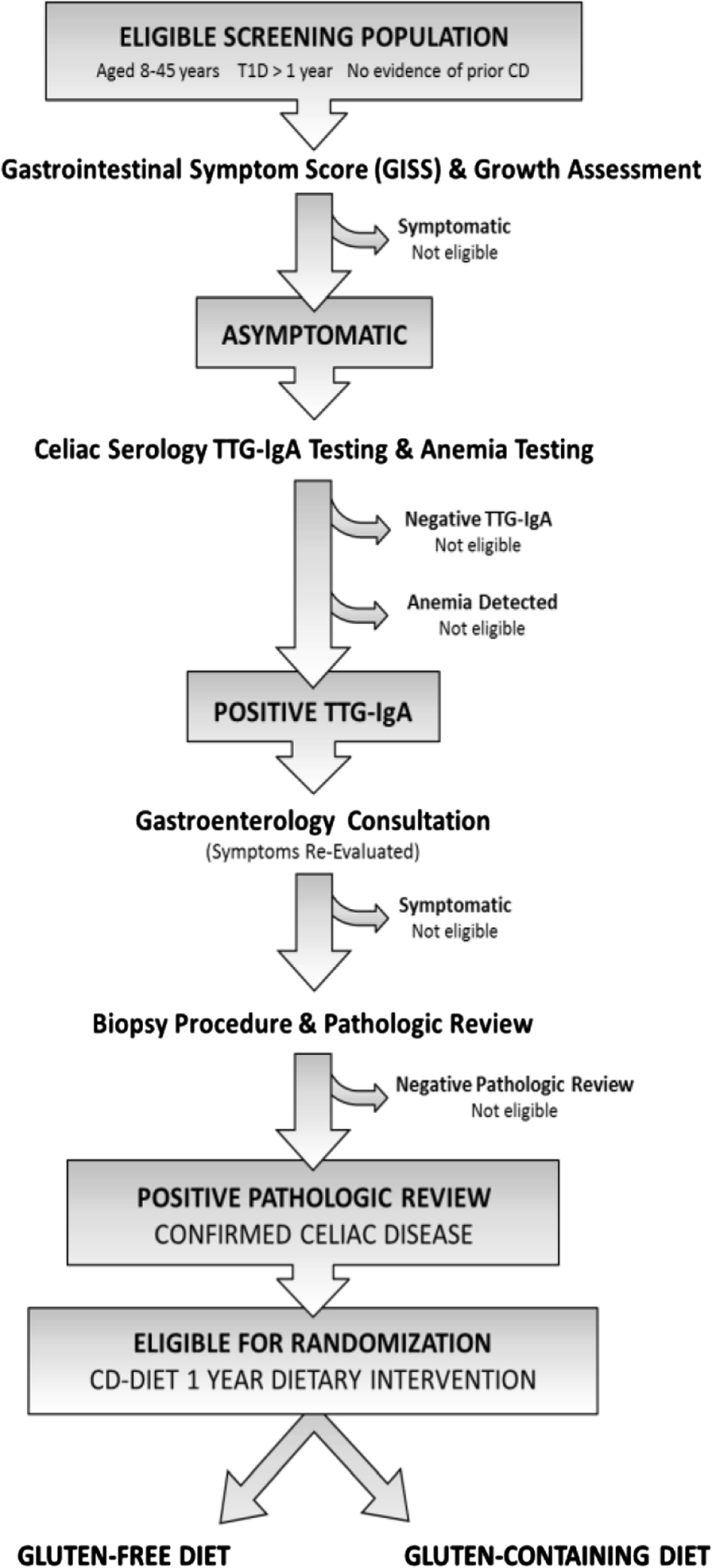
Flow diagram illustrating screening and eligibility for Intervention. Mahmud et al BMJ Open 2015

CD-DIET is designed to evaluate the efficacy and safety of the gluten-free diet (GFD) in patients with asymptomatic biopsy confirmed CD and T1D. Patients (ages 8–45) will be randomly assigned to a GFD or continue with their usual diabetes gluten containing meal plan [27] (Fig. 2 study flow diagram). Patients will be followed-up every 3 months where Visit 1 is randomization, Visits 2,3,4 and 5 as 3, 6,9, and 12 months respectively from randomization. We anticipate that this may be a challenge as adherence to dietary advice is reported to be the lowest of all treatment types [28] with a range of 36-96 % as strict adherence [15].

Designing the dietary protocol takes into account 3 key components to successful adherence to the GFD, as identified by the literature and our experience: understanding of the GFD, ability to maintain a GFD while away from home and membership in a celiac support group [23]. These factors not only are met via the guide developed and described above but have been embedded into the dietary curriculum with opportunities to evaluate knowledge and adherence at every visit. Therefore a robust dietary curriculum which includes a detailed educational plan of what and when information will be relayed, with opportunities for assessment and evaluation of dietary quality, food consumption patterns and adherence, are key elements to the success of this study.

The dietary curriculum is planned to detect any dietary deficiencies and imbalances of macro and micronutrients as well as dietary fiber, calcium, vitamin D and iron using ESHA, a Food Processor with Nutrition Analysis software. The study will also include gluten consumption patterns of the subjects enrolled using the Osborne formula [29].

“Your guide to Well Being: Managing celiac and Diabetes” was designed specifically for the intervention arm (GFD) to address the intricate educational and social needs of both CD and diabetes. It was written in a modular form as an easy to use guide with topics ranging from explaining the diagnosis and managing a GF home to special considerations and travel with T1D. Figure 1 depicts an overview of the dietary curriculum with the study visits.

*The Role of the dietitian in this study is* identified as supporting the subjects to the arm that they are randomized to, but not intended to intensify diabetes management. Subsequently adherence in this study is defined as the ability to follow the prescribed diet. Dietary adherence will be assessed at each clinic visit both objectively and subjectively.

### Components dietary assessment and adherence

3 major components of assessment for adherence were identified which include subjective and objective measures: 1. a comprehensive dietary interview with alternating 3 day food records and typical day recalls. 2. Application of Celiac Dietary Adherence test (CDAT) and 3. Serology –TTG IgA titres.

### Objective measures of assessment

**Fig. 3:**
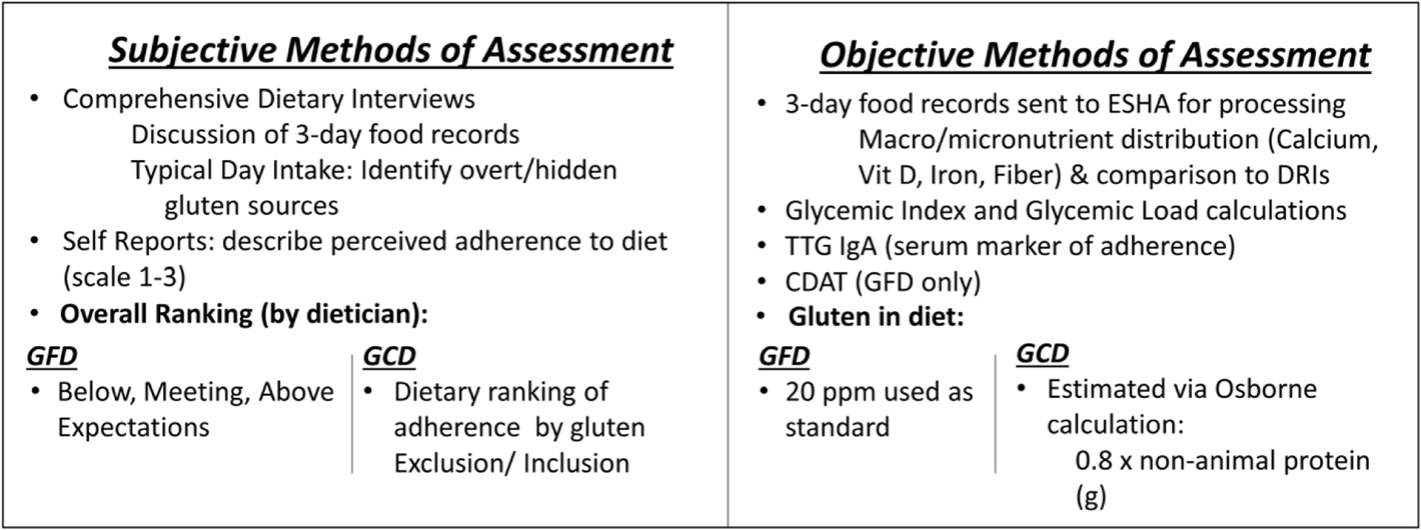
methods of Assessment for GFD/GCD arms

The objective measures in the dietary assessments include (Fig. 3):

1. A 3 day food record will be collected and clarified. Subsequently, records will be sent to central analysis where nutrient intake will be analyzed using the computer based ESHA food processor which contains a wide data base capable of analyzing over 160 food nutrient components and compare to DRIs. The analysis will include macronutrient distribution to be compared to CDA practice guidelines and specific nutrients including calcium, iron, vitamin D and fiber. Alterations in macronutrients, vitamins and minerals have been associated with imbalances and/or deficiencies with the GFD [5–8]. Additionally, Glycemic index (GI) and glycemic load (GL) of mixed meal will be calculated and analyzed for both GFD and GCD.

In this study we are also planning to describe the food consumption patterns of our population with dietary gluten. This will be done by multiplying the plant proteins of overt gluten sources by a factor of 0.8 [29]. This is a relatively novel approach in North America in relation to its application to dietary intake. Our findings from a previous study show that this approach to dietary gluten estimation is reasonable and valid (29). Dietary gluten for the GFD group will be estimated by using the maximum 20 ppm standard as defined by Health Canada’s labelling regulations [30].

2. Celiac dietary Adherence Test (CDAT)- a 7 item questionnaire that has been demonstrated to have a highly predictive value (88 %) for verifying compliance with the GFD in adults, as compared to TTG IgA and dietary evaluation [31]. It will be administered to the subjects randomized to the GFD at every visit after randomization (visits 2–5) (Fig. 4).

**Fig. 4:**
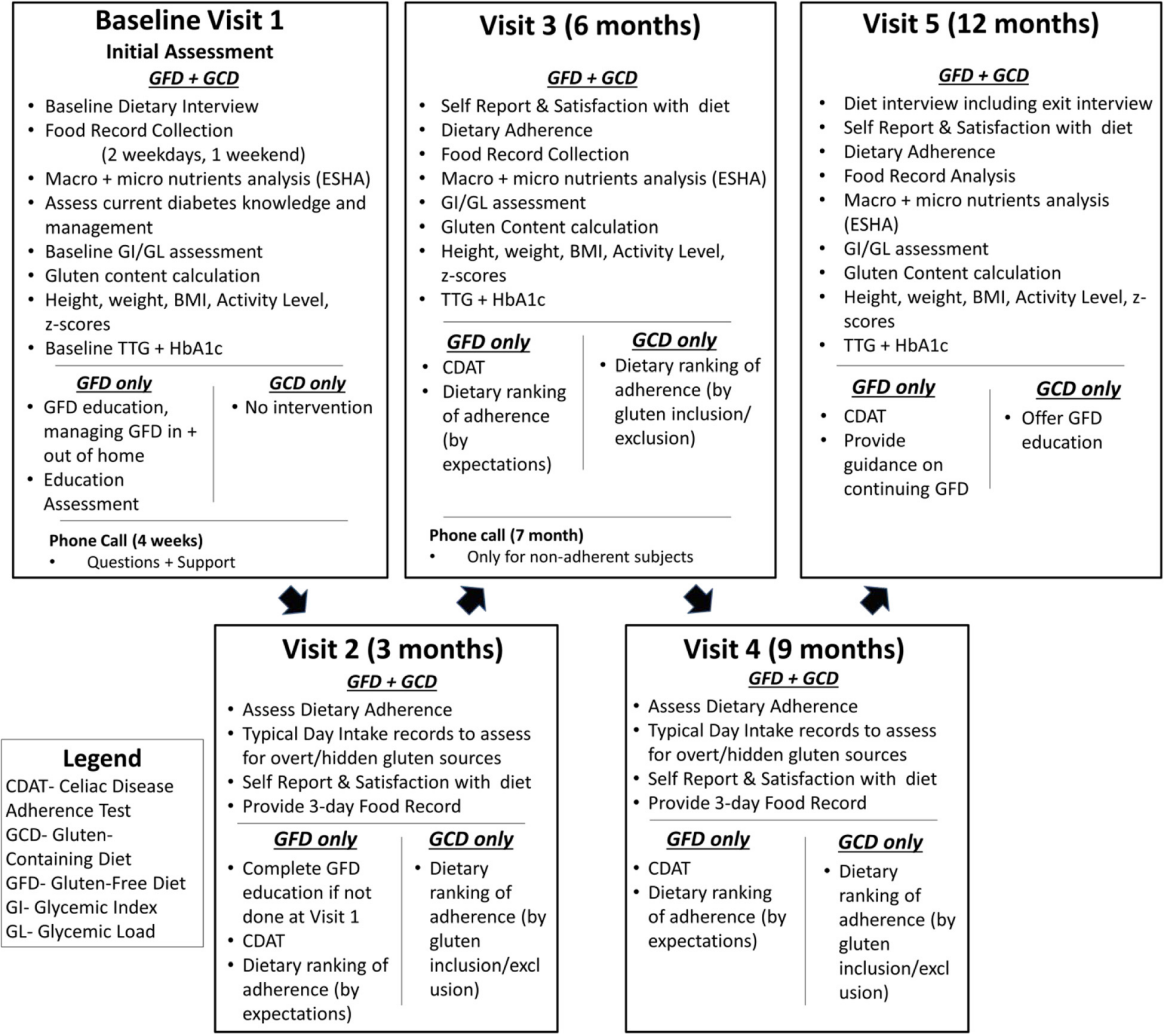
CD-DIET Visit components
3. Tissue Transglutaminase IgA Antibody Assay-TTG IgA – we anticipate that if subjects are adherent, serology will normalize or decrease with subjects randomized to the GFD and remain elevated for subjects randomized to the GCD. Blood sample will be taken from both groups at visits 3 and 5.

### Subjective measures of assessment

The subjective measures in the dietary assessments include (Fig. 3):

1. Comprehensive dietary interviews to also include 3 day food records (at visits 1, 3 and 5) and a typical day recall (at visits 2, 4) (Fig. 1). In the absence of a 3 day food record a typical day recall will be taken. Through the records, the dietitian will assess for the presence of dietary gluten and/or hidden sources of gluten.
2. Ranking: At the end of every dietary interview a global ranking of adherence will be applied by the dietitian. A ranking of ‘expectations’ will be applied to the subjects on the GFD arm. The ranking will range from ‘Below Expectations’ - minimal understanding of GFD concepts and inability to distinguish carbohydrate containing foods, to ‘Meeting Expectations’ – with the GFD and ability carb count, and ‘Above Expectations’ – advanced understanding of the GFD, seeks out additional information about GF carb sources and demonstrates an interest in the curriculum. A ranking of gluten inclusion and exclusion will be applied to the GCD arm. The ranking will range from “Complete inclusion of gluten containing (GC) foods to ‘Some restriction of GC foods’ to a ‘strict restriction of GC foods’.
3. Subject’s own self report: Subjects will also have an opportunity to evaluate their own adherence with the diet that they have been randomized to from ‘strict’ to ‘partial’ to ‘none’ adherent.

## CD-DIET study dietary overview: methods and design

Subjects aged 8 to 45 years old with established T1D and biopsy confirmed asymptomatic CD will be randomized to treatment with GFD for one year or continue with their usual GCD as described (Refer to Figs. 2 and 4) [27].

### Dietitian visits

All subjects on both arms of the study will have five visits with a trained dietitian. Worksheets specific to each arm and visit have been designed to ensure consistent collection of information, and uniform parameters for assessment and education. All worksheets will be filled out by the dietitian at every visit. All participants will fill out food records (two weekdays and 1 weekend day) for visits 1, 3 and 5 (at initial assessment, 6 month and 12 months). Food records will be sent to ESHA, and will be reviewed with the participants at their following visit (visits 2 and 4, 3 and 9 months, respectively). Glycemic Index (GI) and Glycemic load (GL) will be calculated to describe the dietary carbohydrates of each arm using glucose as a reference. GI of mixed meals per day will be ranked as: Low GI ≤55, Medium GI 56–69 and High GI ≥70. Similarly GL per day will be ranked as: Low GL <80, Medium GL 80–120 and a high GL >120 [32]. Dietary gluten evaluation will be performed by using the Osborne formula for all subjects at baseline. Twenty ppm will be applied to the GFD arm as per Health Canada’s definition of GF claims.

A typical day intake is incorporated into the dietary interview at visits 2 and 4 (3 and 9 month visit respectively). All records will be assessed for dietary exclusion or inclusion of gluten. At visits 2–5 (3,6,9, and 12 months) inclusive all participants will complete with the dietitian a 'self-report' questionnaire designed to assess their comfort (both groups) and understanding of their assigned diet.

### Height, weight, BMI and z-scores

Regular monitoring of anthropometric parameters will be performed to assess safety. Height and weight will be obtained at every clinic visit and BMI and z-scores will be derived from those measurements. Level of activity will be ranked as per The Canadian Physical activity guidelines (www.csep.ca/) (Fig. 1).

### Dietary adherence

Dietary adherence will be assessed at each visit following the baseline visit. Adherence assessment will be classified as objective and subjective as described earlier.

Subjective and objective assessment of adherence will include food records collected at visits 1, 3 and 5 (randomization, 6 and 12 months) and a typical day intake at visits 2 and 4 (3 and 9 months) for both groups. All records will be evaluated for inclusion and exclusion of gluten. In addition, food records will be analyzed via ESHA, dietary food processor. GI/ GL and gluten content will be assessed at baseline, 6 and 12 months in both arms. For the intervention group, the adherence to GFD will be further assessed by the application of the Celiac Dietary Adherence Test (CDAT). Additionally, TTG-IgA titers will be measured at 6 (Visit 3) and 12 months (Visit 5) visits to asses GFD/GCD compliance.

After randomization (visit 1) at visits 2 to 5, both study groups, intervention and control, will be interviewed to assess and document adverse events including severe hypoglycemia requiring assistance, hospitalization, any changes in non-insulin medications, supplements and vitamins from previous visit. HbA1c and TTG will be collected at visits 1, 3 and 5 for both arms. GISS and CDAT questionnaires will also be completed only for intervention group, at each visit.

A trained dietitian from the leading site will train identified dietitians from the partner sites using standardized educational material and a detailed manual of operations describing the goals objectives of the dietary curriculum and the steps for the dietary interview at each visit. The standardized tools will be used to maintain quality and consistency of dietary education and collection of information.

#### Intervention group

Visit 1: The dietary interview will consist of a comprehensive nutritional assessment followed by the GFD education. “Your Guide to Well-being: Managing Celiac Disease and Diabetes” is a resource manual, divided into modules, that was specifically designed for this population to address the complex dietary nature of living with both CD and T1D. This resource as well as "Pocket Dictionary of Ingredients" and 'Checking Ingredient List' by the Canadian Celiac Association will be provided to the participants along with the GFD education. Participants will be directed to online resources such as Apps that will help and guide them in determining presence of gluten in food, as well as locations of gluten free restaurants and stores in their vicinity. At the end of the education session an assessment will be carried out using food labels for the comprehension of the GFD, carb counting skills and current diabetes management. Three day food records will be collected, clarified and sent to central analysis for ESHA (Elizabeth Stewart Hands and Associates) processing.

#### Control group

Visit 1: The dietary interview will consist of a comprehensive nutritional assessment including on how well the subjects are managing their current diet and ensuring consumption of GC foods. Food labels will be used to assess carbohydrate counting skills. Three day food records will be collected, clarified and sent to central analysis for ESHA processing.

A 4 week follow-up call will be done in both groups to support, address questions and concerns, document any changes in non-insulin medications, supplements and vitamins and document adverse events including severe hypoglycemia requiring assistance.

### Follow-up visits 2, 3 and 4 (Months 3, 6 and 9)

#### Intervention group

The dietary interview will address any gaps in education; provide nutritional support and reinforcement of GFD topics and modules, as required. Subject's food records analysis from visit 1 will be reviewed for imbalances and deficiencies in macronutrients as per Canadian Diabetes Association guidelines and against DRIs. A plan to address any issues or concerns will be implemented and a “Typical Day Intake” will be performed to assess for gluten exclusion and/or inclusion. Subjects with the dietitian will complete a 'self-report' questionnaire designed to assess their comfort with their assigned diet. The dietitian will rank the subjects adherence using specific criteria, see Fig 3. Three day food records will be provided for visit 3.

#### Control group

The dietitian interview will aim to support current dietary management. Subject's food records analysis from visit 1 will be reviewed for imbalances and deficiencies; a plan to address any issues or concerns will be implemented. A “Typical Day Intake” will be performed to assess for gluten exclusion and/or inclusion. Subjects with the dietitian will complete a 'self-report' questionnaire designed to assess their comfort with their assigned diet. The dietitian will rank the subjects adherence using specific criteria, see Fig 3. Three day food records will be provided for visit 3.

At 3 (Visit 2) and 9 months (Visit 4) a “Typical Day Intake” interview will be performed as part of the diet interview and food records will be provided for next visit. At visit 3 and 5 (6 and 12 months) in addition to the dietary interview, food records will be collected and sent to central analysis for ESHA processing. Handouts specific to each group (GFD/GCD) have been designed to address the possible dietary deficiencies or imbalances in macronutrients as well as fiber, iron, calcium and vitamin D.

### Conditional telephone contact (Month 7)

This telephone call will apply to subjects who are found to be non-adherent (as per TTG IgA results from Visit 3) to their assigned diet. For both groups: dietitian will address follow-up questions and concerns, barriers to dietary adherence, document any changes in insulin regimen or in non-insulin medications, supplements and vitamins, document adverse events including severe hypoglycemia requiring assistance.

### Visit 5 (Month 12)

For both arms this visit will entail all of the details as per visit 3 (month 6) visit. The dietitian will conduct an exit interview consisting of 7 questions for GFD and 4 questions for the GCD. Subjects assigned to the GCD will be asked if they would like to follow the GFD.

### Outcome assessments

#### CDAT and TTG and Dietitian’s ranking

We anticipate that the subjective ranking by the dietitian will correlate with the subject’s TTG, CDAT and self-report in both arms. We also anticipate that the average GI and the GL content of the GFD will be higher than the GCD due to the nature of some of the GF foods which tend to be more refined higher in carbohydrate and contain less fiber than the GC equivalents. The difference in GI and GL between the groups may also explain the possible BG variability in both groups as will be documented with (continuous glucose monitoring (CGM)).

### Changes in dietary intervention assignment (cross-overs)

In cases of symptom presentation as defined by Mahmud et al. [28] subjects will be crossed over into the GFD.

Power calculation and statistics:

The outcome measure used to calculate sample size will be the change in HbA1c level from baseline to month 12. Based on previous work, in a non-randomized comparison of HbA1c levels over time in type 1 diabetes patients with asymptomatic CD who received a GFD versus patients with type 1 diabetes alone, an increase in HbA1c of 8.3 ± 1.1 % to 8.9 ± 1.5 % was observed.10 As such, 91 evaluable individuals per group are required to provide 80 % power to detect a difference of at least 0.5 % at the 0.05 level of significance. The enrolment of 200 participants will allow for a non-evaluable rate of about 10 %. To reach the recruitment target of 200 individuals, approximately 5000 patients with an established diagnosis of type 1 diabetes will be screened for asymptomatic CD. Efficacy analyses will be performed on the intent-to-treat population consisting of all randomized participants.

The primary efficacy outcome, the change in HbA1c at 12 months, will be analyzed using an analysis of covariance (ANCOVA) model that includes adjustment for baseline HbA1c, age groups and investigative sites. Treatment effect will be quantified using the point estimate, the two-sided 95 % CI, and associated p value. Significant effect will be declared at the 5 % significance level.

Severe hypoglycemic episodes over time will be analyzed as Poisson counts using the generalized estimating equations approach to account for correlation among repeated measures within an individual. The QOL data over time will be analyzed by means of a linear mixed effects model including factors for treatment, investigative sites, age groups, and baseline QOL scores. BG variability and change in Z-score at the lumbar spine will be analyzed using the ANCOVA approach. The Statistical Analysis System (SAS) procedures GENMOD, MIXED and GLM in SAS V.9.2, will be used to perform the respective analyses.

Patient consent: Obtained.

Ethics Approval: Research Ethics Board Hospital for Sick Children, University of Toronto and at all recruitment sites.

The CD-DIET study data safety and monitoring board will perform regular reviews of collected safety and efficacy data. The protocol was peer reviewed by the JDRF.

### Trial organization

- JDRF-CCTN (Juvenile Diabetes Research Foundation – Canadian Clinical Trials Network) – financial and organizational support
- Robarts Clinical Trials and Sickkids – Monitoring, safety and adverse events coordination
- Group Organization – Paediatric and adult endocrinologists, gastroenterologists, diabetes dietitians, and research coordinators in each of different sites.

## Discussion and dissemination

The CD-DIET study is a large scale clinical trial that will further contribute to the pool of knowledge and novel implications to the treatment of asymptomatic celiac disease and TID. The dietary curriculum will be coordinated across all centers by the main site dietitian, using standardized educational materials and a manual of operations. Both pediatric and adult patients will be randomized to a GFD or GCD. Adherence to the diets will be determined from the dietary interview, TTG-IgA blood tests and CDAT (GFD) and subjects’ self-report. CDAT is highly correlated with Standardized Dietician Evaluation (SDE) and even superior to TTG IgA serology with regards to GFD adherence [31]. The Canadian context to the study is important, as the 2013 Canadian Diabetes Association’s clinical practice guidelines suggests a targeted symptom based screening approach [33]. While the literature supports the implementation of a GFD in symptomatic CD and T1D patients, it is less clear as to the benefits for asymptomatic patients. Our curriculum was established in concurrence with the current literature and is designed to address a number of issues: the limited educational literature the challenges associated with dietary quality and adherence and the implicated social challenges of a GF lifestyle in addition to T1D and. Participants will be supported by dietitians throughout the study and subjects on the intervention group and later the control group will have to have access to ‘Your Guide to Well-being: Managing Celiac disease and Diabetes' a resource specifically designed to address the nutritional intricacies and related challenges of both conditions: CD and T1D.

The dietary quality of the GFD will be assessed through food records and interviews. The literature reports that GF equivalents tend to be lower in micronutrients and fibre, but higher in calories, fat and sodium [5, 13]. The participant’s diets will be evaluated for deficiencies and imbalances of macronutrients and micronutrients. We will assess the quantity and quality of carbohydrate intake using the glycemic index as well as glycemic load and calculate dietary gluten in our subject population. Dietary gluten quantification was previously demonstrated in Europe and applied to one of our smaller pilot studies with some limitations inherent to methodology and with the Canadian and American nutrient databases. We have described a method to help apply the Osborne calculation in North America [11] and plan to correlate adherence to gluten consumption and describe our population’s consumption patterns as it relates to CD and T1D.

Participants will also have the opportunity to report their adherence to the diet that they are randomized to through a ‘self-report’ questionnaire from ‘strict’ to ‘partial’ to ‘non’ adherent [34]. Although some studies show that patients overestimate adherence to GFD and that reliability of self-reports decreases with time on a GFD [16, 35], our previous findings show a close correlation between self-reports and dietitians’ assessment [36].

One of the greater challenges with asymptomatic patients if that they report better QOL and health prior to diagnosis [25]. This is likely due to the perceived burden of novel dietary restriction.

This study will answer some of the questions with regards to perceived benefits of GFD, especially when almost a third [37] of health practitioners are unsure whether they should be recommending GFD to asymptomatic patients [38]. The study was initiated in 2012, now active at 16 centers, and it is anticipated that results will be available by 2016.

## Abbreviations

CCA: Canadian Celiac Association
CD: Celiac Disease
CDA: Canadian Diabetes Association
CDAT: Celiac Dietary Adherence Test
CD-DIET Study: Celiac disease and Diabetes- Dietary **I**ntervention and Evaluation Trial
CPG: Clinical Practice Guidelines
GCD: Gluten containing diet
GFD: Gluten free diet
GISS: Gastrointestinal symptom scale
HbA1c: Glycosylated hemoglobin
PedsQL: Pediatric Quality of Life
QOL: Quality of life
SDE: Standardized Dietitian Evaluation
T1D: Type 1 Diabetes
TTG: Tissue Transglutaminase antibodies
TTG - IgA: Tissue Transglutaminase IgA Antibody Assay
